# Development of a dynamic motion platform with two independent drive systems for radiotherapy

**DOI:** 10.1002/acm2.13971

**Published:** 2023-03-23

**Authors:** Masahide Saito, Hideyuki Kawakami, Toshihiro Suzuki, Hidekazu Suzuki, Koji Ueda, Hikaru Nemoto, Naoki Sano, Hiroshi Onishi

**Affiliations:** ^1^ Department of Radiology University of Yamanashi Yamanashi Japan; ^2^ APEX medical Inc. Tokyo Japan; ^3^ Department of Radiology Kasugai‐CyberKnife Rehabilitation Hospital Yamanashi Japan

**Keywords:** lung cancer, motion phantom, motion tracking, radiotherapy, two drive system

## Abstract

**Background:**

There are some motion platforms for radiotherapy quality assurance. However, no platform with two drive systems that can move along three axes is available.

**Purpose:**

The purpose of this study is to develop a dynamic motion platform with two drive systems capable of three‐axis motion and to evaluate its motion performance.

**Methods:**

The developed moving platform had two drive systems that use the same equipment. Each axis of the platform used can support a maximum load of 10 kg. The motors for moving the platform in each direction are capable of a drive stroke up to 40 mm. The drive speed is 30 mm/s at maximum load fluctuation. To evaluate the static positional accuracy of this system with an arbitrary input movement, the XYZ position of each axis was measured using a coordinate measuring machine operating from 0 to 40 mm at 10 mm intervals. In addition, the accuracy of dynamic motion was verified with Sine waveform inputs of different patterns to the three axes for approximately 60 s, and they were compared with the resulting detected signals by SyncTrax.

**Results:**

The two drive systems were successfully operated on three axes by using independent control systems. For static position, the accuracies were within 0.2 mm, 0.05 mm, and 0.14 mm for lateral, longitudinal, and vertical directions, respectively. For dynamic motion, the mean absolute errors in the X, Y, and Z axes between the platform inputs and SyncTrax detected signals were 0.14 ± 0.10 mm, 0.16 ± 0.12 mm, and 0.16 ± 0.11 mm, respectively.

**Conclusions:**

A new dynamic platform for radiation therapy with two drive systems capable of three‐axis motion was developed, and the positional accuracy of the drive axes was confirmed to be less than 0.2 mm.

## 1 INTRODUCTION

For radiotherapy on organs with respiratory movement, establishing a validation system before administering treatment is crucial. Traditionally, various dynamic motion platforms and phantoms have been used to evaluate the effects of respiratory movement, including the CIRS dynamic phantom,^1^ which simulates the thorax and can read any respiratory waveform, thereby allowing the target area to move in three dimensions. The CIRS platform can also be validated using an individual phantom and a detector.^2^ QUASER (Modus Medical Devices) is another device that has been used for quality assurance of four‐dimensional computed tomography (4DCT) scan techniques in multicenters.^3^ For this device, a new device that can be used under MR has recently been introduced.^4^ In addition, Delta4 Hexa‐motion has been developed as a platform for 3D detectors and is used for the verification of moving objects.^5^

All phantoms are capable of translational three‐axis movement of the target part; however, they are only capable of the ventral–dorsal one‐axis movement of the surrogate part. For example, the CIRS phantom is capable of three‐axis translation of the target part with rotation, whereas the surrogate part has only one axis of translation. However, there is no phantom with two drive systems that can move along three axes, that is, no phantom can also move along three axes for the surrogate part. The development of such phantoms will enable dynamic verification under more complex surrogate motions. For example, while tracking a tumor using a gold marker implanted in the body, if the distance between the tumor and gold marker is large because of the migration of the gold marker^6^, ^7^ and if the tumor and gold marker movements are slightly different, a phantom with two drive systems that can reproduce complex movements is necessary for dynamic verification. In addition, for irradiation using external signals such as surface‐guided radiotherapy (SGRT), the position of the body surface may move slightly in any other direction apart from the anterior‐posterior.^8^, ^9^ If a phantom takes three‐dimensional movement into account, more accurate dynamic verification may be possible in above situation.

Therefore, in this study, we developed a dynamic motion platform with two drive systems capable of three‐axis motion and evaluated its motion performance.

## 2 MATERIALS AND METHODS

### 2.1 Dynamic motion platform

We have developed a dynamic motion platform with two drive systems that is capable of three‐axis motion and can also read arbitrary waveforms. The platform can use basic mathematical waveforms (Sin and Cos waveforms) and signals extracted from humans at 0.03 ms intervals. However, these waveforms must be prepared by the user as comma‐separated value (CSV) files, and these files can be loaded up to about 10 h. For single‐drive system movements, movements up to ±40 mm can be realized for all three axes. However, for two drive system movements, the larger the movement, the greater the possibility of interference between the two drive systems depending on the starting position and movement pattern. Therefore, it is necessary to verify the interference situation of each axis before use.

**FIGURE 1:**
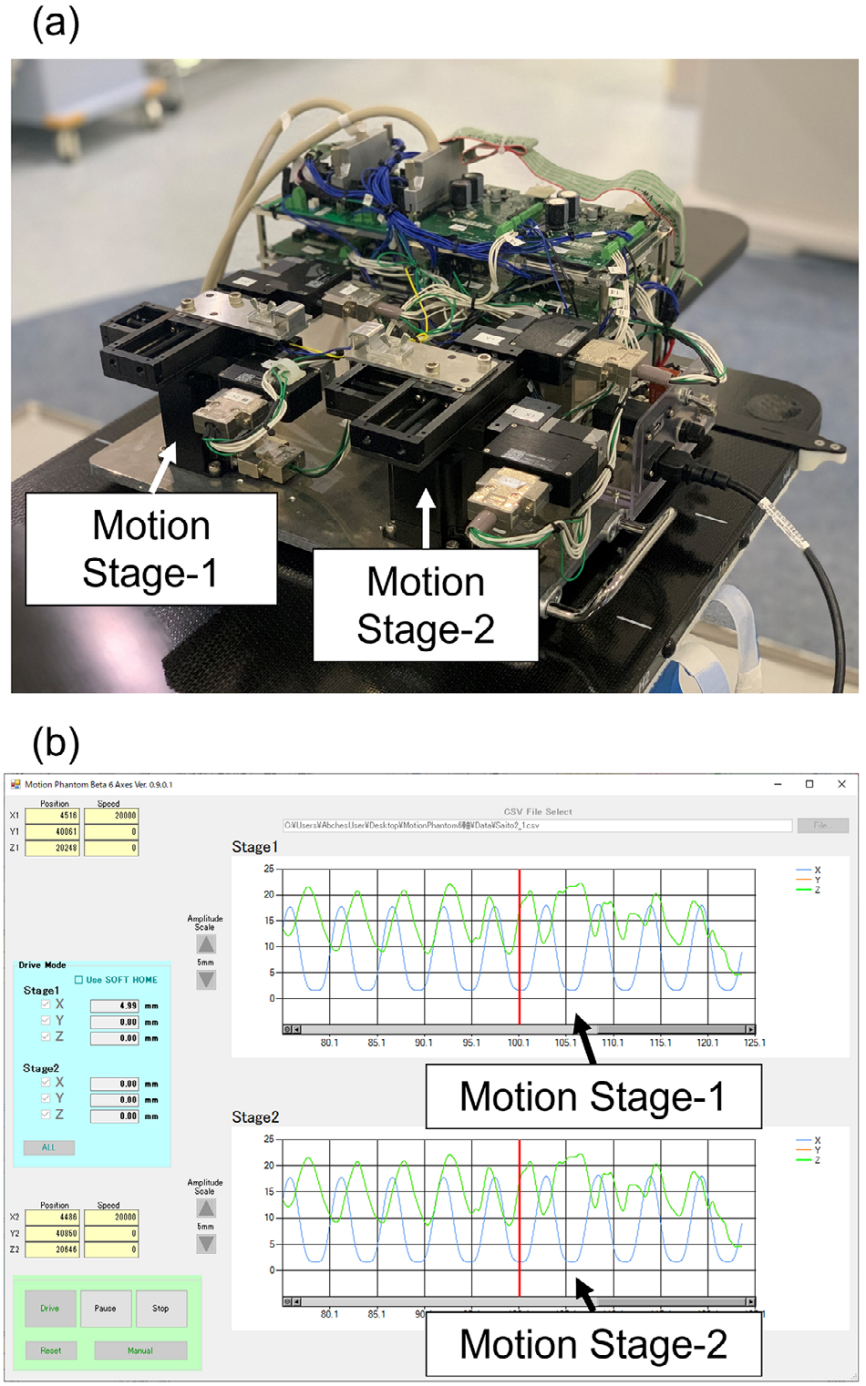
Interface of the dynamic motion platform. (a) Platform constructed with motion Stages 1 and 2. (b) Software developed for importing waveforms.

Figure 1a presents an overview of the developed dynamic motion platform. It is characterized by two drive systems that use the same equipment. The weight of the system is 13.4 kg and it is connected to a computer for operation. Each axis of the platform supports a maximum load of 10 kg. ZABER LMR050A‐T3 (Zaber Technologies Inc., Vancouver, Canada) was used as the motor for the X‐(lateral) and Y‐(longitudinal), and ZABER VSR40A‐T3 (Zaber Technologies Inc., Vancouver, Canada) was used as the motor for the Z‐axis. Both motors were capable of a drive stroke of up to 40 mm, and the drive speed was 30 mm/s at maximum load fluctuation. Figure 1b shows an overview of the software developed to read arbitrary waveforms in CSV format for the two drive systems. The software works well on Windows 10 or 11. Static movement is also possible, and any waveform can be continuously read. Arbitrary start positions can be entered for each axis, and functionalities such as start, pause, and stop can be manually controlled. Respiratory waveforms are displayed in the upper and lower rows for each of the axes, respectively (Figure 1b upper row: Stage‐1, Figure 1b lower row: Stage‐2).

**Areas of Expertise**

TH‐ External beam‐ photons: Motion management—interfraction

TH‐ External beam‐ photons: Motion management—intrafraction

TH‐ External beam‐ photons: cyberknife

TH‐ External beam‐ photons: extracranial stereotactic/SBRT

### 2.2 Static positioning evaluation

**FIGURE 2:**
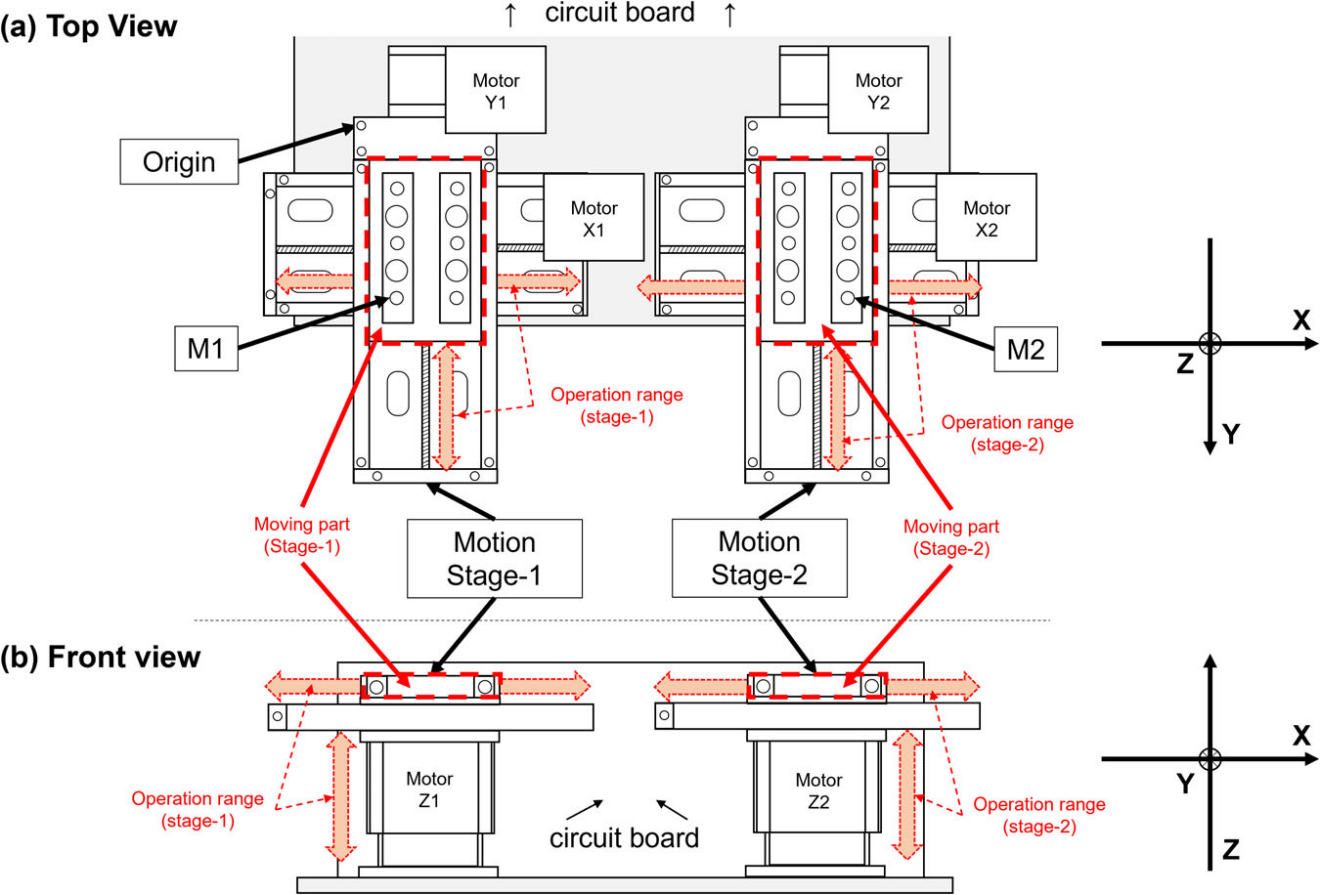
Details of the moving platform structure. (a) Top and (b) front views. The measurement points are denoted as M1 and M2 based on the origin point in (a).

This study entailed the verification of the basic positional behavior of the two drive systems. The measurement points are shown in Figure 2. A structurally defined point on the fixed part of Stage 1 was set as the measurement origin, and the measurement points on the movable parts of Stage 1 and Stage 2 were set as M1 and M2, respectively. For M1 and M2, the XYZ position of each axis was measured using a coordinate measuring machine (CRYSTA‐PLUS M544, developed by Mitutoyo, Kanagawa, JAPAN) that operated from 0 to 40 mm at 10 mm intervals. The measurement accuracy was less than 10 μm in all directions. Further details are mentioned in the white paper.^10^

### 2.3 Dynamic positioning evaluation

In this study, the accuracy of the dynamic movement of the drive system was verified using SyncTraX® FX4 (Shimadzu, Kyoto, Japan). SyncTraX® FX4 is the new system used for tracking irradiation,^11^ and in recent years, some technical^12^, ^13^ and clinical^14^ evidences have validated its efficacy. In this verification, the driving accuracy of only one of the drive systems was tested (Stage‐1 only), assuming that both drive systems in the platform are equivalent in their accuracy because they have a similar structure. Here, dynamic drive accuracy was verified by inputting typical sine waveforms with different patterns to all the three axes for approximately 60 s and comparing them with the results detected by SyncTrax. Three metal markers were placed on the platform and position accuracy was checked on the X‐ray image of the SyncTraX system with a detection cycle of approximately 60−70 ms.

## 3 RESULTS AND DISCUSSION

The actual movement of the dynamic platform is described in video 1. The two drive systems were successfully operated on three axes by using independent control systems. Tables 1 and 2 show the driving accuracy results for Stages 1 and 2, respectively. The vertical axis represents the measurement value obtained by the coordinate measuring machine, and the horizontal axis represents the set movement amount of the platform. For the X‐axis, the accuracy was within 0.2 mm when the platform moved from 0 to 40 mm. However, it was also moved systematically with respect to the Y‐axis, by approximately 0.13 mm as the X‐axis moved. Solely for the Y‐axis, the same accuracy of 0.05 mm or lower was confirmed; however, the X‐axis was driven with accuracies of 0.08 mm each as the Y‐axis moved. As regards the Z‐axis an accuracy of 0.14 mm or lower, with almost no movement of the other axes, was achieved. The aforementioned characteristics were common to Stage 1 and Stage 2.

**TABLE 1:** Result of the driving accuracy for Stage 1.

	Measured value [mm]			
Magnitude of movement [mm]	Move axis	X	Y	Z
0	X‐axis	0.000	0.000	0.000
	Y‐axis	0.000	0.000	0.000
	Z‐axis	0.000	0.000	0.000
10	X‐axis	10.189	0.133	0.000
	Y‐axis	0.018	10.007	0.000
	Z‐axis	−0.002	0.009	10.132
20	X‐axis	20.196	0.261	0.000
	Y‐axis	−0.094	19.949	0.000
	Z‐axis	−0.006	0.010	20.113
30	X‐axis	30.147	0.416	0.000
	Y‐axis	−0.268	30.019	0.000
	Z‐axis	−0.007	0.031	30.132
40	X‐axis	40.149	0.509	0.000
	Y‐axis	−0.380	40.017	0.000
	Z‐axis	−0.010	0.040	40.133

**TABLE 2:** Result of the driving accuracy for Stage 2.

	Measured value [mm]			
Magnitude of movement [mm]	Move axis	X	Y	Z
0	X‐axis	0.000	0.000	0.000
	Y‐axis	0.000	0.000	0.000
	Z‐axis	0.000	0.000	0.000
10	X‐axis	9.938	0.090	0.000
	Y‐axis	−0.138	9.964	0.000
	Z‐axis	0.000	0.000	10.102
20	X‐axis	20.009	0.221	0.000
	Y‐axis	−0.308	19.930	0.000
	Z‐axis	−0.004	0.002	20.095
30	X‐axis	29.961	0.293	0.000
	Y‐axis	−0.396	29.970	0.000
	Z‐axis	−0.012	0.018	30.091
40	X‐axis	39.986	0.466	0.000
	Y‐axis	−0.543	39.931	0.000
	Z‐axis	−0.019	0.046	40.084

**FIGURE 3:**
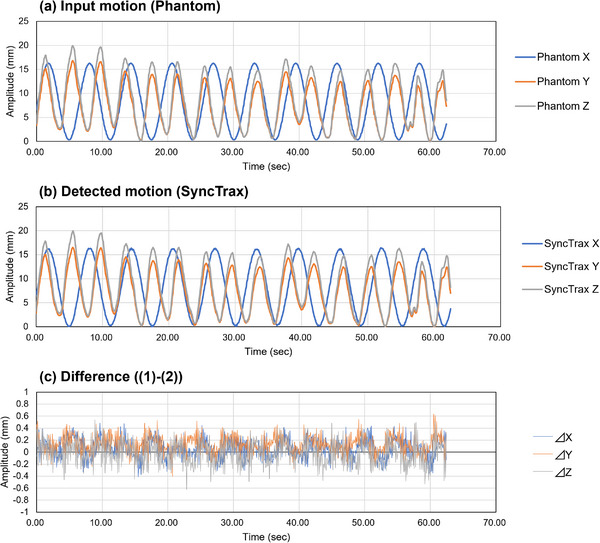
Results of dynamic movement verification: (a) platform input values (in X, Y, and Z directions). (b) SyncTrax detection signals in each direction. (c) Difference between them.

The results of the dynamic movement verification is shown in Figure 3: Figure 3a is the respiratory waveform input to the dynamic platform, Figure 3b is the signal detected by SyncTrax, and Figure 3c is the difference between the two waveforms. The mean absolute errors in the X, Y, and Z axes were 0.14 ± 0.10, 0.16 ± 0.12, and 0.16 ± 0.11 mm, respectively.

**FIGURE 4:**
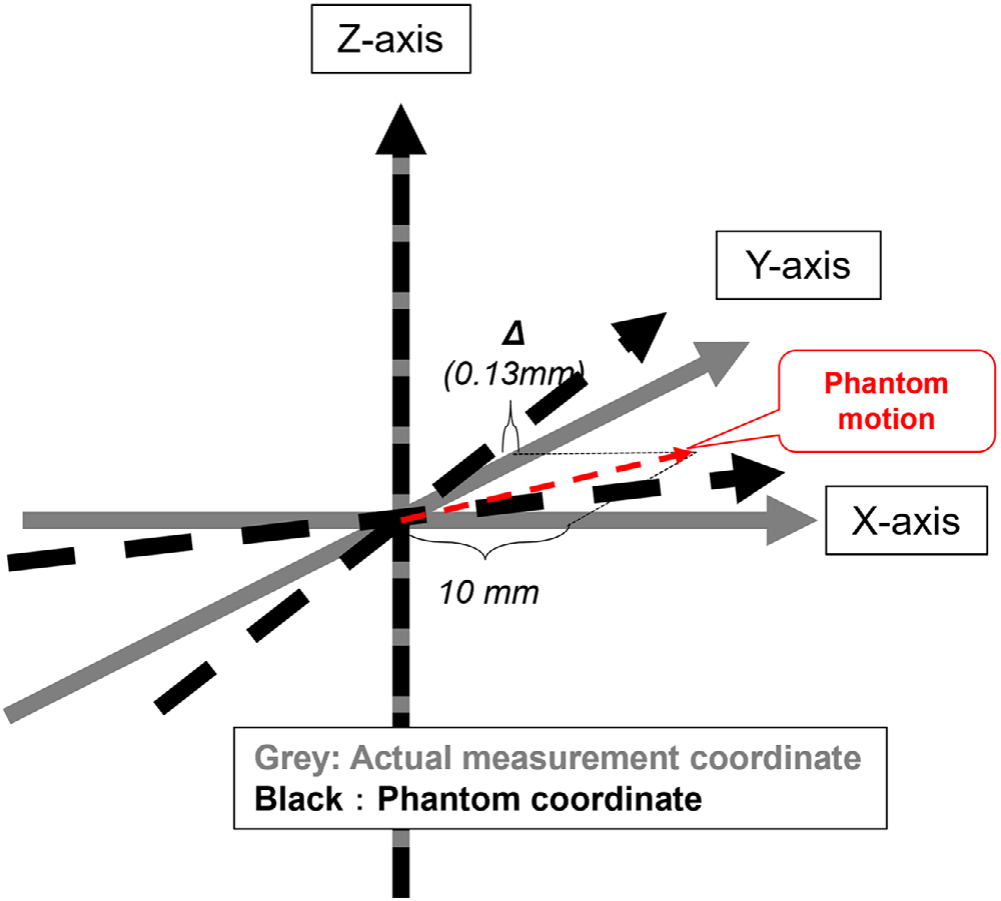
Coordinate system used in this study. The gray arrows show the actual measurement coordinates, and the black arrows show the platform coordinates. The red dashed line shows the systematic shift in the Y‐direction when the platform was moved 10 mm in the X‐direction.

Figure 4 shows the coordinate systems employed in this study. The gray lines denote the exact coordinate system, and the black lines show the coordinate system of the platform. The results indicate that the X‐Y plane was slightly tilted while maintaining a right angle. However, because the magnitude of movement on the Y‐axis was approximately 0.15 mm when the platform was moved 10 mm on the X‐axis.

As a comparison with other commercially available products in terms of accuracy, Table 3 shows the accuracy values of the CIRS phantom, QUASAR phantom and our platform. The accuracy achieved in this study is confirmed to be comparable to that of available products. However, it should be kept in mind that these measurement conditions are slightly different.

**TABLE 3:** Comparison of the driving accuracy of our platform with those of other motion phantom.

Motion phantom	Accuracy
CIRS dynamic motion phantom^1^	±0.10 mm (Motion Accuracy)
QUASAR™ MRI^4D^ Motion Phantom^2^	±0.25 mm (Motion Precision, [along Z‐axis])
Our platform	±0.20 mm (Static and Motion Accuracy [average value], X, Y, Z‐axis)

It should be noted that the maximum speed of our platform is 30 mm/s. Therefore, it is difficult to reproduce conditions such coughing; it is suitable for verification only under free‐breathing conditions. Furthermore, direct irradiation of approximately 500 Gy to the platform body (circuit board) may cause system malfunctions. Therefore, especially during non‐coplanar beam validation, caution must be used to avoid exposure to large amounts of radiation.

**FIGURE 5:**
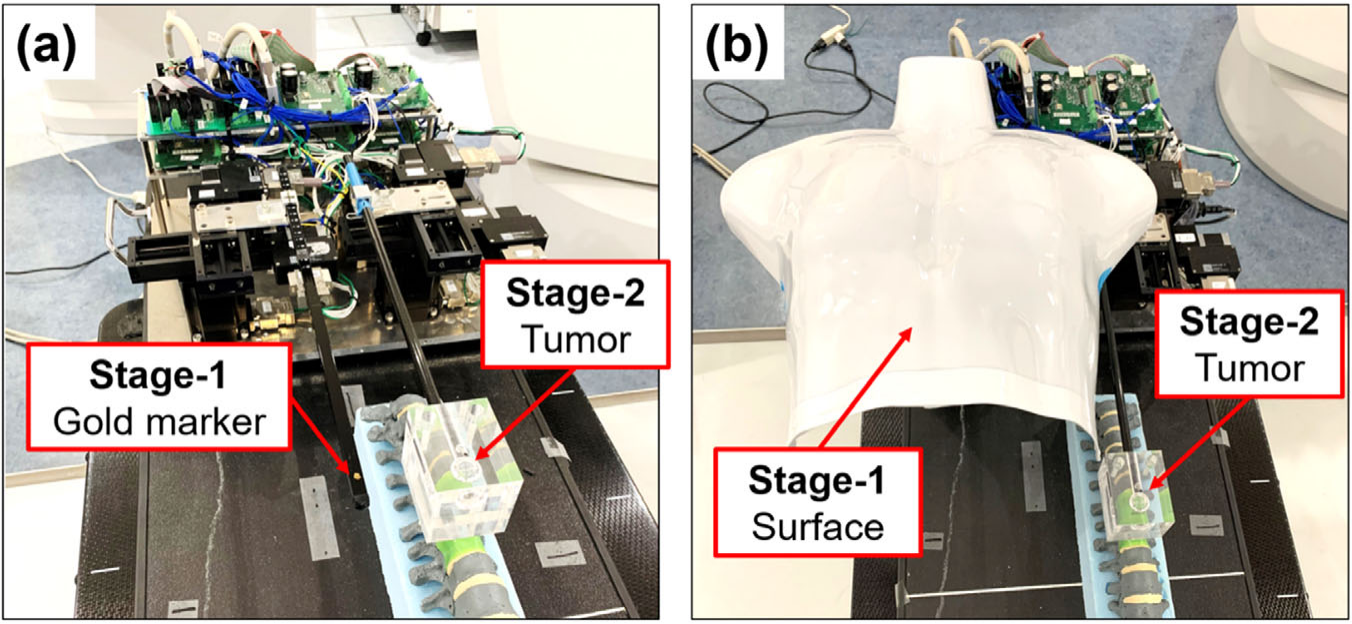
Examples of the platform use: (a) Example to simulate the movement of a gold marker inside the body and the movement of a tumor. (b) Example to simulate the movement of the body surface and the movement of a tumor.

The phantom developed in this study may be used in the following practical cases. Examples of typical platform uses are shown in Figure 5. First, Figure 5a shows one example to simulate the movements of a gold marker and tumor inside the body. This phantom may be useful for verification of tumor tracking when the marker cannot be implanted at the intended position (i.e., locations away from the tumor, or locations that do not seem to correlate with movement, etc.). In addition, Figure 5b shows another example to simulate the movements of the body surface and tumor; however, the body surface has a particularly complex motion not only in the anterior‐posterior direction, but in various other directions as well.

One limitation of this study is that it does not consider the effects of the installation and fixing accuracy of the actual installation jig or the effects of the load capacity (up to 10 kg, according to the specifications). Further, specific verification regarding rotational movements was not performed. Therefore, in actual use, the accuracy achieved may be worse than the results presented herein, and further verification according to the setting conditions will be necessary.

## 4 CONCLUSION

A novel dynamic platform for radiation therapy having two drive systems and capable of three‐axis motion was developed in this study; the positional accuracy of the drive axes was confirmed to be less than 0.2 mm.

## AUTHOR CONTRIBUTIONS

Study conception and design: Masahide Saito, Hideyuki Kawakami, Naoki Sano, and Hiroshi Onishi. Acquisition of data: Masahide Saito, Hideyuki Kawakami, Toshihiro Suzuki, Hidekazu Suzuki, and Koji Ueda. Analysis and interpretation of data: Masahide Saito, Hideyuki Kawakami and Hikaru Nemoto. Drafting of manuscript: M.S., and Hiroshi Onishi. Critical revision: Masahide Saito, Hideyuki Kawakami Naoki Sano, and Hiroshi Onishi.

## CONFLICT OF INTEREST STATEMENT

This study was supported by joint research with APEX Medical Inc. and Hamano Engineering.

## Supporting information

Supporting Information

## Data Availability

The data that support the findings of this study are available from the corresponding author upon reasonable request.
